# Integrated support vector regression and an improved particle swarm optimization-based model for solar radiation prediction

**DOI:** 10.1371/journal.pone.0217634

**Published:** 2019-05-31

**Authors:** Hamidreza Ghazvinian, Sayed-Farhad Mousavi, Hojat Karami, Saeed Farzin, Mohammad Ehteram, Md Shabbir Hossain, Chow Ming Fai, Huzaifa Bin Hashim, Vijay P. Singh, Faizah Che Ros, Ali Najah Ahmed, Haitham Abdulmohsin Afan, Sai Hin Lai, Ahmed El-Shafie

**Affiliations:** 1 Department of Water Engineering and Hydraulic Structures, Faculty of Civil Engineering, Semnan University, Semnan, Iran; 2 School of Energy, Geoscience, Infrastructure and Society, Department of Civil Engineering, Heriot-Watt University, Putrajaya, Malaysia; 3 Intitute of Energy Infrastructure (IEI), Department of Civil Engineering, Universiti Tenaga Nasional, Selangor, Malaysia; 4 Department of Civil Engineering, Faculty of Engineering, University of Malaya, Kuala Lumpur, Malaysia; 5 Department of Biological and Agricultural Engineering, Zachry Department of Civil Engineering, Texas A&M University, College Station, Texas, United States of America; 6 Department of Environmental and Green Technology, Malaysia-Japan International Institute of Technology (MJIIT), Universiti Teknologi Malaysia (UTM), Kuala Lumpur, Malaysia; Northeast Electric Power University, CHINA

## Abstract

Solar energy is a major type of renewable energy, and its estimation is important for decision-makers. This study introduces a new prediction model for solar radiation based on support vector regression (SVR) and the improved particle swarm optimization (IPSO) algorithm. The new version of algorithm attempts to enhance the global search ability for the PSO. In practice, the SVR method has a few parameters that should be determined through a trial-and-error procedure while developing the prediction model. This procedure usually leads to non-optimal choices for these parameters and, hence, poor prediction accuracy. Therefore, there is a need to integrate the SVR model with an optimization algorithm to achieve optimal choices for these parameters. Thus, the IPSO algorithm, as an optimizer is integrated with SVR to obtain optimal values for the SVR parameters. To examine the proposed model, two solar radiation stations, Adana, Antakya and Konya, in Turkey, are considered for this study. In addition, different models have been tested for this prediction, namely, the M5 tree model (M5T), genetic programming (GP), SVR integrated with four different optimization algorithms SVR-PSO, SVR-IPSO, Genetic Algorithm (SVR-GA), FireFly Algorithm (SVR-FFA) and the multivariate adaptive regression (MARS) model. The sensitivity analysis is performed to achieve the highest accuracy level of the prediction by choosing different input parameters. Several performance measuring indices have been considered to examine the efficiency of all the prediction methods. The results show that SVR-IPSO outperformed M5T and MARS.

## Introduction

Solar energy is one of the most important forms of energy. Although fossil fuels can produce a large amount of energy, they cause various kinds of pollution [1,2]. Undoubtedly the knowledge of solar radiation is important as it has direct or indirect impact on the current and future life [3]. This energy affects the agriculture, industry engineering, health and the tourism sector of any nation [2].

Solar radiation (SR), however, does not cause environmental pollution [3,4]. Solar energy can be converted to heat energy or electricity [5,6] and has high potential as an energy supply in various fields. Solar energy can be accessed easily and is not limited to specific regions in the world. Low maintenance cost is one of the most important features of solar energy [7]. Recently, there have been many advances in solar energy generation, such as solar cells [8,9]. The pyranometers or actiongraphs are used for the direct measurement of solar radiation [8]. The measurements can be very accurate with the new sensors, but also the device cost, installation and the maintenance cost to bear is a big drawback for many countries.

Different researchers have used mathematical and statistical methods and artificial intelligence techniques to compute solar radiation energy [3,8]. A combination of artificial intelligence and soft computing with mathematical and statistical methods has led to the development of mathematical models. Increased precision, speed, and ease of computation are considered desirable features for techniques based on artificial intelligence [10]. Statistical regression models have weaknesses that could be overcome with the incorporation of optimization algorithms. For example, SVR could be integrated with PSO to optimally adapt SVR’s unknown parameters instead of using a trial-and-error procedure that could lead to non-optimal selection of the unknown parameters and, as a result, poor prediction accuracy.

### Background

Artificial neural network (ANN) models were used by [11] to predict SR. The ANN model was compared with SVR, and it was found that multi-layer perceptron neural networks could achieve better RMSE than could SVR and other kinds of ANNs. Latitude, longitude, monthly minimum and maximum temperatures, and relative humidity were used as inputs. Landeras et al. [12] used genetic programming (GP) to compute SR. A radial neural network with four input layers, namely, maximum temperature, hours of sunshine, relative humidity, and minimum temperature, was used. The results based on GP had a higher correlation coefficient than the results of an ANN trained by PSO [13]. The number and structure of the neurons and hidden layers of the ANN model depended on the PSO. The results showed that the improved ANN was more accurate than the classic ANN model with simple architecture. Khatib et al. [14] compared different methods of computing SR and showed that regression methods had drawbacks in terms of identifying the unknown parameters and that artificial intelligence methods generally outperformed them. The unknown parameters of the regression methods were computed by optimization. K-means clustering and ANNs were used by [15] to compute SR. A comparison of the results showed that the clustering method had a smaller RMSE and MAE than the ANN model. SR has also been estimated by a hidden Markov model and a generalized fuzzy model [16].

Different combinations of meteorological parameters have been considered for predicting the SR. Atmospheric pressure, relative humidity, wind speed, and sunshine were used for the SR simulation. The best results were achieved by the combination that used all the inputs except wind speed. The ANN was improved by a genetic algorithm (GA) for computing SR [17]. The GA optimization method computed the number of hidden layers and neurons in the ANN. The results showed that the predictive ability of the ANN was related to the training algorithm and input combinations. A tree regression model and an ANN were used to predict daily global SR (DGSR) for two locations [18]; the results showed that the ANN estimated DGSR satisfactorily. Mohammadi et al. [19] studied SR based on the combination of SVM and a wavelet method. Air temperature, humidity, and sunshine duration were considered as inputs. The new hybrid SVM achieved more accurate results than simple SVM and ANN models. Olatomive et al. [20]trained a neural network for the computation of solar radiation by computing the number of hidden layers, and the results were compared with those of GP and SVR. The results showed that the trained neural network reduced the RMSE value by 20 and 25%, respectively, compared to the GP and SVR. Premalatha et al. [21]used the Levenberg-Marquardat algorithm, resilient propagation and the scaled conjugate gradient for the development of a neural network and the simulation of solar radiation; the results showed that the mean absolute error of the Levenberg-Marquardat algorithm was 20 and 25% less than those of the resilient propagation and scaled conjugate gradient methods, respectively.

SR has been predicted by a least squares support vector machine (LSSVM) and the firefly optimization algorithm (FFOA) [22] by functionalizing the latter to optimally select the unknown parameters for the LSSVM. The results showed that the new hybrid structure achieved smaller RMSE than did GP and SVR. The accuracies of the three methods (the adaptive neuro-fuzzy interface system (ANFIS), SVR and ANN) were considered for computing SR [23]. The results showed that ANFIS with inputs of the daily maximum and minimum temperatures, hours of sunshine, and rainfall, exhibited better performance than other models. Ibrahim and Khatib [24] used the hybrid structure of Random Forest and Firefly algorithm for the computation of solar radiation; the results showed that the hybrid method had a lower value for the error index than the GP and neural network methods. Meenal et al. [25] used SVR and ANN to simulate solar radiation; the results showed that these models could reduce the RMSE by 20 and 25% over the empirical models. Kumar et al. [26] simulated solar radiation with the different neural networks, and the radial basis neural network had the best results in comparison to other kinds of neural networks; the MAE and RMSE values were negligible for the radial basis neural network. Voyant et al. [27] reviewed several methods for the simulation of solar radiation in the literature; the results showed that the kind of inputs and the accurate estimation of unknown parameters in the regression methods had important effects on the results. Wang et al. [28] trained a system of fuzzy rules using the particle swarm algorithm for the computation of solar radiation and compared the results to those of a neural network and genetic programming. This showed that the improved fuzzy rules increased the correlation coefficient between the observed data and simulated data in comparison to the neural network and GP methods.

Alfadda et al. [29] used the k nearest neighbours and the SVR method to determine solar radiation based on hours of sunshine, maximum temperature, minimum temperature and relative humidity; the results showed that the k nearest neighbours could reduce the RMSE by 20% in comparison to SVR.

Wang et al. [30] used radial basis neural network (RBNN), generalized regression neural network (GRNN) and multilayer perceptron neural network (MPNN) for estimating of solar radiation. The results indicated that MPNN and RBNN can predict more accurately compared with the GRNN and there is significant different among these models.

Rohani et al. [31] used the Gaussian progress regression with K fold cross validation for the estimating of daily solar radiation. The results proved that the new model can be used with small size of data group and it can predict better than empirical model.

However, the general results of the literature review showed that neural networks and fuzzy methods have good performance in estimating solar radiation but require the accurate determination of several parameters, such as weights or the number of hidden layers in a neural network [32,33]. In addition, the structures of these methods are more complex than that of regression methods would be if the regression methods could be applied to solar radiation.

### Innovation and objectives

A literature review shows that SVR has been widely used for SR simulation [22,32–36]. Defining the best values of the regression parameters is important for regression models, as this step influences the final prediction accuracy for the whole process. Most of the previous studies determined the best values of the regression parameters using trial and error [22,27,35,37]. Conventionally the integration was basically developed on the basis two different concepts. The first one is to initialized the regularization parameters (three parameters) of the SVR and then identify the best fitness of these parameters by trials and errors for different group of these random initialization. On the other hand, the second concept was developed as modifications for the first concept by initializing the SRV regularization parameters within a predetermined domain to accelerate the training process and by adding k-fold procedure for avoiding the over-fitting for the model performance.

In the current research, the concept of the integration between the SVR and PSO is principally different than the previous ones, as it was developed to compute the objective function for the Root Mean Square Error (RMSE) for the initialized regular parameters of the SVR to search for their optimal values of the initial parameters which are considered as multiple decision variables using PSO algorithm.

In addition, the present study suggests a novel structure for an SVR model that is integrated with Improved PSO (IPSO) as an optimizer. IPSO determines the best values of the regression parameters in SVR, and then the SVR model is used to compute SR. The literature shows that PSO has high potential for use in different optimization applications, such as image processing, dam and reservoir operation, mathematical functions, hydrological prediction, and optimal design [36,38–41]. The new version of algorithm is defined for this paper so that the global ability search for the PSO algorithm increase and the algorithm can escape from local optimums well and thus, a new operator for the algorithm is defined to update the global solutions. Another innovation aspect of the current paper is related to the comprehensive evaluation of different models for the solar estimation in the different climates.

The objectives of this paper are i) to evaluate the ability of a new version of SVR to predict SR, ii) to compare the new method to the M5T, GP and multivariate adaptive regression models, and iii) to investigate the effect of different input variables on the models’ predictive ability. Two case studies in Turkey are used to validate the proposed prediction model.

## Methods

### Support vector regression

For SVR, a linear function is defined that the related independent and dependent variables. A linear equation is used as the main equation in SVR and is expressed as

$$f\left(x\right)=W^{Tr}.x+b$$ (1)

where *x* is the input variable, *W* is the weighting vector, *b* is the bias, *Tr* is the transpose, and *f(x)* is the output variable. Vapnik et al. [42] suggested the following error function to prevent an overfitting deficit. The function is defined based on the following equation and is known as the epsilon intensive function:

$$\left|y-f\left(x\right)\right|=\left\{\begin{array}{l} 0\leftarrow if\left(y-f\left(x\right)\right)\leq \kappa \\ \left|y-f\left(x\right)\right|-\kappa =\xi \end{array}\right\}$$ (2)

is subject to

$$\begin{array}{l} \left(w_{i}.x_{i}+b\right)-y_{i}<\kappa +\xi_{i}^{+}\\ y_{i}-\left(w_{i}.x_{i}+b\right)\leq \kappa +\xi_{i}^{-}\\ \xi_{i}^{+},\xi_{i}^{-} \end{array}$$ (3)

where $\xi_{i}^{+}$ and $\xi_{i}^{-}$ are violations of the *ith* training data that are below and above (0*κ*,+*κ*), *κ* is the permissible error threshold, *y*_*i*_ is the output variable, *x*_*i*_ is the input variable, *w*_*i*_ is the weight vector, *ξ* is the computed penalty for the estimated error, and *b* and *w* are two decision variables. The values of *b* and *w* are computed when the SVR completes the training level. The values of *b* and *w* are inserted into Eq (1), and *f(x)* is computed. There are several kernel functions that can convert linear Eq (1) into nonlinear forms. The radio kernel function has been widely used in previous articles [22,27,35,37].

$$f\left(x\right)=w^{Tr}.K\left(x,x_{i}\right)+b$$ (4)

$$K\left(x,x_{i}\right)=\text{exp}\left(-\frac{{\left|x-x_{i}\right|}^{2}}{2\gamma^{2}}\right)$$ (5)

where *K*(*x*, *x*_*i*_) is the kernel function and *γ* is a parameter. The most important duty of SVR is to compute the values of the parameters *κ* and *γ*. Huang and Wang [43] found that these parameters have an important effect on the results.

### Particle Swarm Optimization

The basis of PSO is the group behaviour of particles in a search space. In addition, members of the community can profit from the experiences of other members. An important feature of the PSO algorithm is social behaviour, in that it directs members towards the best place in the search space. Each particle in PSO is known as one solution candidate in the domain of possible solutions. The *ith* member of the population is represented by a D-dimensional vector *X*_*i*_ = (*x*_*i*1_, *x*_*i*2_, .., *x*_*id*_)^*T*^. In addition, *V*_*i*_ = (*v*_*i*1_, *v*_*i*2_, .., *v*_*id*_)^*T*^ is the velocity of the particle. The best previously computed position of the *ith* particle is *P*_*i*_ = (*p*_*i*1_, *p*_*i*2_, .., *p*_*iD*_)^*T*^, and the index *g* in the equations indicates a global guide for the particle in the population. The positions and velocities of the particles are updated based on the following equations:

$$v_{id}^{n+1}=\chi \left[iwv_{id}^{\text{n}}+c_{1}r_{1}^{n}\frac{\left(p_{id}^{n}-x_{id}^{n}\right)}{\Delta t}+c_{2}r_{2}^{n}\frac{\left(p_{gd}^{n}-x_{gd}^{n}\right)}{\Delta t}\right]$$ (6)

$$x_{id}^{n+1}=x_{id}^{n}+\Delta t\left(v_{id}^{n+1}\right)$$ (7)

Here, *d* = 1,2.., *D*, *χ* is a constriction coefficient, *iw* is the inertia weight, *c*_*1*_ and *c*_*2*_ are acceleration coefficients, *r*_*1*_ and *r*_*2*_ are random parameters, and Δ*t* is the time interval.

The global best particle is considered as solution candidate, it guides the other particle toward to the other neighbours, and thus, this issue can cause that the particles trap in the local optimums. As a result, many particles will not have chance of a comprehensive search of large problem space. Thus, an effective strategy can cause the particles to get rid of local optimums and a global strategy, based on following equation, is used for improving the model’s efficiency.

$${p^{\prime }}^{n}{}_{gd}=p_{gd}^{n}\times \left(1+\lambda \times N\left(0,1\right)\right)$$ (8)

Where, *λ*: disturbance factor, *N*(0, 1): the normal distribution. *λ* was tested with the different values (i.e. 0.01, 0.02, 0.05, 0.10, 0.15 and 0.2) and there is not significant in the results and thus, it was considered 0.1. the $p_{gd}^{n}$ is replaced with ${p^{\prime }}^{n}{}_{gd}$ when the ${p^{\prime }}^{n}{}_{gd}$ has the better value compared to the $p_{gd}^{n}$.

### SVR and IPSO

**Fig 1:**
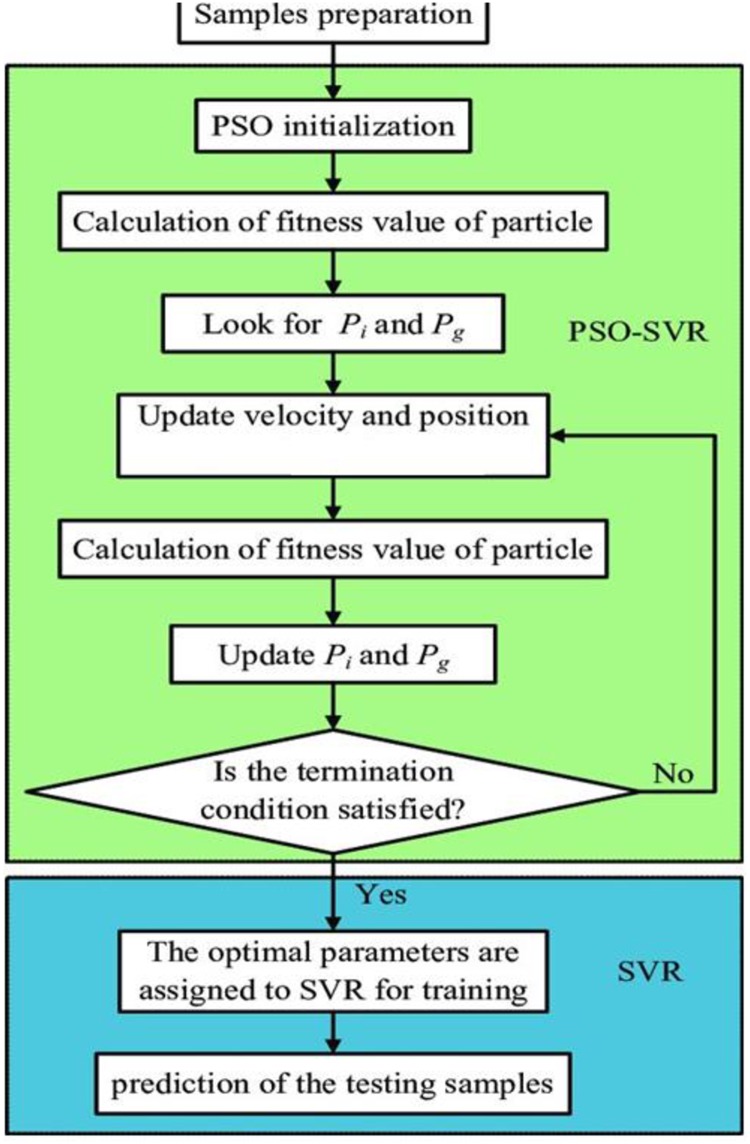
Structure of the hybrid SVR and IPSO.

The hybrid structure of SVR and IPSO was considered through the following steps:

1. Determine the input variables for data collection and processing.
2. Consider the initial values of the SVR parameters.
3. Consider the training level of the SVR and compute the objective function (RMSE) for the input variables.
4. If the stopping criterion is satisfied, the optimal values of the coefficients are extracted for the test level. Otherwise, the algorithm goes to the next level.
5. The parameters are considered decision variables and are inserted into the IPSO. The velocities and positions of these variables are updated, and the algorithm returns to the third step. Fig 1 shows the performance flowchart for SVR-IPSO.

### Genetic programming

**Fig 2:**
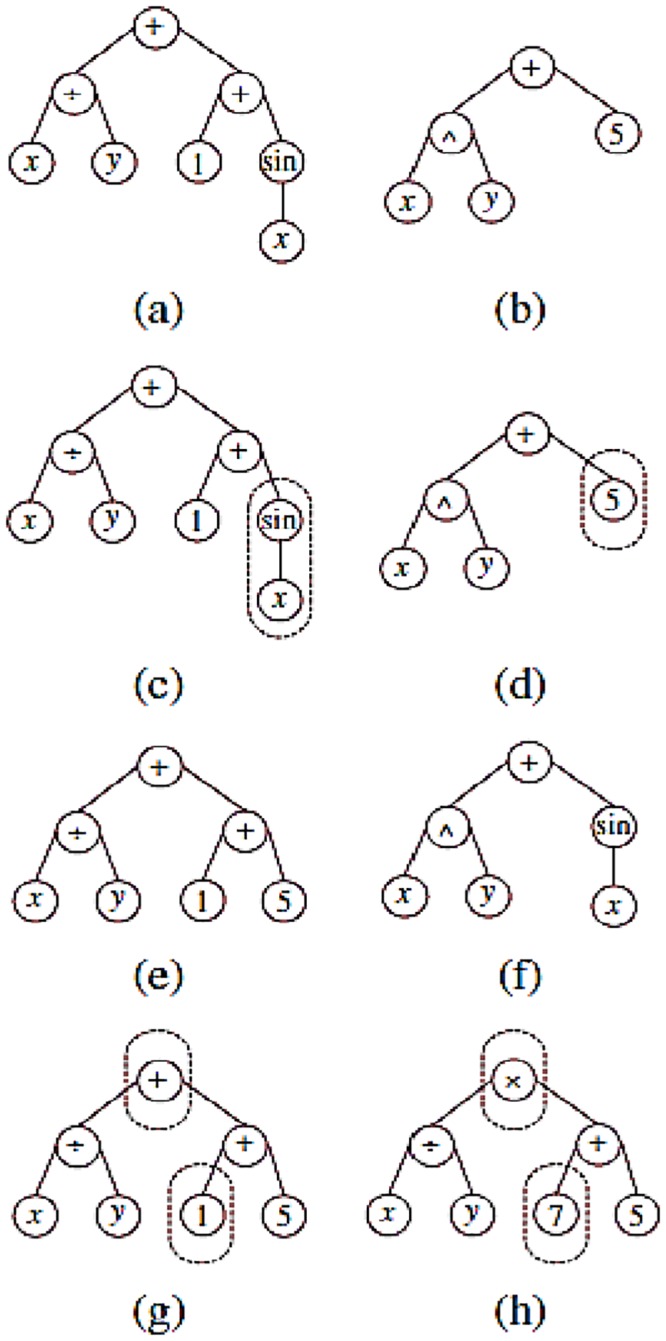
Genetic porgramming structure and search process.

Genetic programming is a successful and widely used method in hydrological simulation. The method searches for a good relationship between the input and output variables. Fig 2b and 2c shows that the method acts based on tree structures. There are different nodes and several branches that connect them to one another. The terminal and function sets are used in the nodes. The terminal sets consist of numerical and non-numerical variables, and the function sets consist of automatic operators (± × ÷), mathematical functions (e.g., sin, cos), Boolean operators and logical expressions. The search process proceeds by generating random trees. Each tree has an objective function with a corresponding error function. A ranking method is used for the selection of trees with better objective functions. Crossover and mutation operators prepare the trees for the next iteration, as shown in Fig 2d and 2e. Two trees are designed, and some branches are considered for them. In addition, the swapping of parent subtrees can be used to generate two new trees. Fig 2d and 2f show the condition of two new trees after crossover. A mutation operator is another genetic operator that exchanges nodes using a random variable or operator. Fig 2g and 2h shows the condition of trees before and after mutation. Two arithmetic operators (± and ×) and three mathematical functions (sin, cos and power (x^y^)) are considered for the GP; a Gaussian membership function has been shown to give good results for solar radiation [19,44].

## Case study

**Fig 3:**
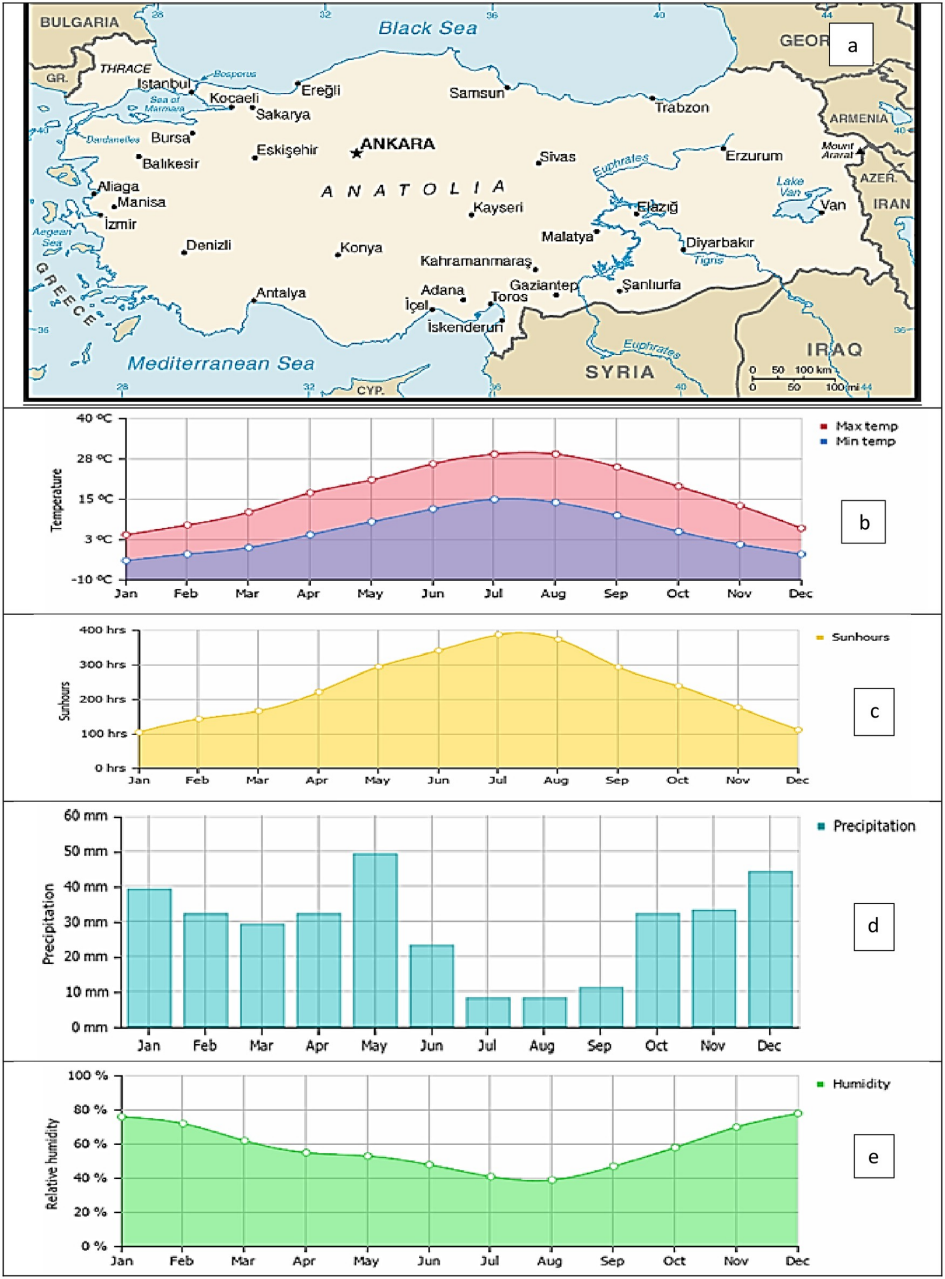
a) Location of the studied stations b: Temperature Average c: Sun hours Average, d: Rainfall average and e: Humidity average.

The present study deals with computing solar radiation in a Mediterranean region of Turkey. The Adana and Antakya stations are considered (Fig 3). The major climatic features of this region are inclination towards rainy winters and hot summers. The Adana station is located at latitude 37.22°N, longitude 35.40°E, and an altitude of 20 m; Antakya is located at latitude 36.22°N, longitude 35.40°E, and an altitude of 20 m. The climatic conditions of the region are affected by a winter season with high rainfall as well as hot summers. The SR distribution shows that the region has high solar energy potential and Turkey has high potential for solar energy because it is in the northern hemisphere. The most value for solar radiation for two stations are observed in July. The annual solar radiation for Antakya is 10.89 MJ/m^2^/day and it is 12.23 MJ/m^2^/day for Adana station.

The data is provided by the Turkish State Meteorological services of Ministry of Agriculture and Forestry, turkey [45]. Data were collected from 1981 to 2016. Based on previous studies, 75% of the data were used for training and 25% for testing [22,27,35,37]. Yearly rainfall varies from 580 to 1300 mm. There are many stations in turkey measure solar radiation based on Siap, Muller and Fuess actiongraphs and there are 11 other stations that have pyranometers for measuring the solar radiation.

Table 1 shows the statistical data for the stations. The highest skew distribution is related to the wind speed, followed by the relative humidity and maximum temperature for both stations. The highest correlation coefficient is for hours of sunshine; thus, there is a high correlation between the hours of sunshine and solar radiation. The necessary data were collected at a geophysics institute for the Antakya and Adana stations from 1981 to 2016.

**Table 1:** Monthly statistical parameters of each climatological data set.

Station	Data set	Unit	x_mean_	x_min_	x_max_	Correlation with SR
Adana	T_max_	°C	31.32	17	43.8	0.802
	T_min_	°C	9.32	-6.4	23.4	0.794
	H_s_	H	223	0	365	0.899
	W_s_	m/s	1.34	0.10	2.3	0.277
	R_h_	%	66.4	46.4	80.8	0.152
	S_r_	Langley	110	36.3	195	1.000
Antakya	T_max_	°C	30.10	14.4	42.6	0.798
	T_min_	°C	9.38	-4.6	24.8	0.818
	H_s_	H	229	29.6	384	0.923
	W_s_	m/s	3.15	1.1	7.1	0.865
	R_h_	%	69.8	49.4	85.1	-0.104
	S_r_	Langley	98.6	27.5	179	1.000

T_max_ = maximum temperature, T_min_ = minimum temperature, H_s_ = sunshine duration, W_s_ = wind speed, R_h_ = relative humidity, S_r_ = solar radiation, x_min_ = minimum value, x_max_ = maximum value and x_mean_ = average value

## Model structure and performance indicators

Evolutionary algorithms, such as IPSO, have parameters whose best values can be reported based on a literature review or experimentation. We set an interval for the random parameters and evaluate the variations in the objective function for various values of the parameters. Then, the best values of the parameters (c_1_ and c_2_ = 2, w = 0.6 and population size for particle swarm = 40) are selected when the objective function converges to its minimum value [38,39,46]. A sensitivity analysis is considered for determining the most suitable parameters and the variation of objective function values is observed accordingly. In this regard, the least objective function value was preferred.

Several scenarios based on different inputs have been proposed based on the correlation coefficients between different input variables and solar radiation. Therefore, four scenarios based on four different input combinations were considered in this study. Keshtgar et al. [3] reported the same inputs for the two stations over the same period (1981–2016).

1. Maximum and minimum temperature
2. Maximum temperature, minimum temperature and sunshine duration
3. Maximum temperature, minimum temperature, sunshine duration and wind speed
4. Maximum temperature, minimum temperature, sunshine duration, wind speed and relative humidity

Keshtgar et al. [3] estimated solar radiation by the M5Tree model (M5T) and multivariate adaptive regression splines using the same inputs. The results were then compared with the results of previous studies. The M5T divided the search space into subspaces and developed a linear regression model for each one [3]. The M5T model divided the data into several sub-collections and then generated decision trees. Each tree had a node on the top and branches connected to other nodes.

The division rule relies on decreasing the standard deviation of the category values that reach a node as an error index for that node [3]. Piecewise linear splines were used in a multivariate adaptive regression model (MARS) known as nonlinear and non-parametric regression. The method could model nonlinear relationships between dependent and independent variables.

To evaluate and examine the performance of the proposed prediction model, several performance indicators have been calculated. The following indices were used to evaluate the developed models:

Root mean square error (RMSE) as the objective function:

$$RMSE=\sqrt{\frac{\sum_{i=1}^{N}{\left(SRm_{i}-SRo_{i}\right)}^{2}}{N}}$$ (9)

Mean absolute error (MAE):

$$MAE=\frac{\sum_{i=1}^{N}\left|SRm_{i}-SRo_{i}\right|}{N}$$ (10)

Mean bias error:

$$MBE=\frac{1}{N}\sum_{i=1}^{N}\frac{SRm_{i}-SRo_{i}}{SRo_{i}}$$ (11)

Nash-Sutcliff efficiency:

$$NSE=1-\frac{\sum_{i=1}^{N}{\left(SRm_{i}-SRo_{i}\right)}^{2}}{\sum_{i=1}^{N}{\left(SRo_{i}-SR_{mean}\right)}^{2}}$$ (12)

Here, *Srm*_*i*_ is the estimated solar radiation, *SRo*_*i*_ is the observed solar radiation, *SR*_*mean*_ is the average observed solar radiation, and *N* is number of data points.

## Results and discussion

### Antakya station

Table 2 shows the performance of different methods in the test stage for the Antakya station. The MAE index shows that the fourth input combination resulted in the best performance for SVR-PSO among all input combinations. The MAE index for SVR-PSO (4) was 43, 37, and 16% less than those for SVR-PSO (1), SVR-PSO (2) and SVR-PSO (3), respectively. The other indices supported this finding. For example, the RMSE index for SVR-PSO (4) was 52, 28 and 8.79% less than those for SVR-PSO (1), SVR-PSO (2) and SVR-PSO (3), respectively. Thus, increasing the number of input data points for SVR-PSO led to improved results for Antakya station.

**Table 2:** Comparison of statistical indices for different methods for estimation of solar radiation in Antakya station.

Method	Input variables	MAE (Langley)	RMSE (Langley)	MBE	NSE	Time (s)
SVR-IPSO (1)	T_max_, T_min_	9.01	19.10	0.141	0.72	10
SVR-PSO (1) *κ* = 0.05, *C* = 61, *γ* = 0.167	T_max_, T_min_	9.12	19.11	0.142	0.701	10
M5T (1) [3]	T_max_, T_min_	17.83	23.80	0.176	0.639	12
MARS (1) [3]	T_max_, T_min_	16.75	21.74	0.166	0.699	14
GP(1)	T_max_, T_min_	16.55	20.72	0.154	0.654	11
SVR-IPSO (2)	T_max_, T_min_, H_s_	8.12	12.52	0.101	0.843	12
SVR-PSO (2) *κ* = 0.05, *C* = 60, *γ* = 0.166	T_max_, T_min_, H_s_	8.25	12.54	0.103	0.842	12
M5T (2) [3]	T_max_, T_min_, H_s_	12.09	15.58	0.106	0.873	15
MARS (2) [3]	T_max_, T_min_ `, H_s_	11.17	14.10	0.112	0.845	17
GP (2)	T_max_, T_min_ `, H_s_	8.29	12.59	0.107	0.844	14
SVR-IPSO (3)	T_max_, T_min_, H_s_, W_s_	6.01	10.11	-0.022	0.942	16
SVR-PSO (3)	T_max_, T_min_, H_s_, W_s_	6.12	10.12	-0.023	0.917	16
M5T (3) [3] *κ* = 0.05, *C* = 55, *γ* = 0.164	T_max_, T_min_, H_s_, W_s_	9.45	12.58	-0.037	0.899	18
MARS (3) [3]	T_max_, T_min_, H_s_, W_s_	9.88	11.56	-0.097	0.915	20
GP (3)	T_max_, T_min_, H_s_, W_s_	6.72	10.45	-0.022	0.824	17
SVR-IPSO (4)	T_max_, T_min_, H_s_, W_s_, Rh	4.10	9.01	-0.020	0.954	18
SVR-PSO (4) *κ* = 0.04, *C* = 54, *γ* = 0.167	T_max_, T_min_, H_s_, W_s_, Rh	5.12	9.23	-0.021	0.924	19
M5T (4) [3]	T_max_, T_min_, H_s_, W_s_, R_h_	9.35	12.35	-0.024	0.903	22
MARS (4) [3]	T_max_, T_min_, H_s_, W_s_, R_h_	9.26	11.12	-0.086	0.921	25
GP (4)	T_max_, T_min_, H_s_, W_s_, R_h_	5.55	9.39	-0.025	0.922	20

SVR-IPSO was compared to M5T, MARS and SVR-PSO models for the Antakya station. The results showed that the improved PSO and SVR predicted better compared to the other models. For example, RMSE for SVR-IPSO (4) is 20%, 57%, 55% and 25% less than SVR-PSO (4), M5T (4), MARS (4) and GP (4). Also, the best input combination for the SVR-IPSO is the fourth combination. Also, the NSE coefficient for the SVR-IPSO performed better than the other methods.

SVR-PSO was compared to M5T and MARS for the Antakya station. The results showed that SVR-PSO outperformed MARS and M5T models. For example, the MBE for SVR-PSO (4), the best SVR-PSO model, was 12 and 75% less than those for M5T (4) and MARS (4), which were the best MARS and M5T models. The NSE for SVR-PSO (4) was 0.924, which was 0.3 and 2.2% greater than those for MARS (4) and M5T (4), the best MARS and M5T models. Table 2 also shows that the results of SVR-PSO are better than those of genetic programming (GP). For example, the MAE and RMSE for SVR-PSO (4) are 7.7 and 1.7%, which are less than those for GP. The best combination of inputs for GP occurs in GP (4); the results show that this GP performs better than MARS and M5T for all inputs. For example, the MAE and RMSE for GP (4) are 66 and 15%, which are less than the MARS indices. An increase in inputs to SVR-IPSO results in good performance.

**Fig 4:**
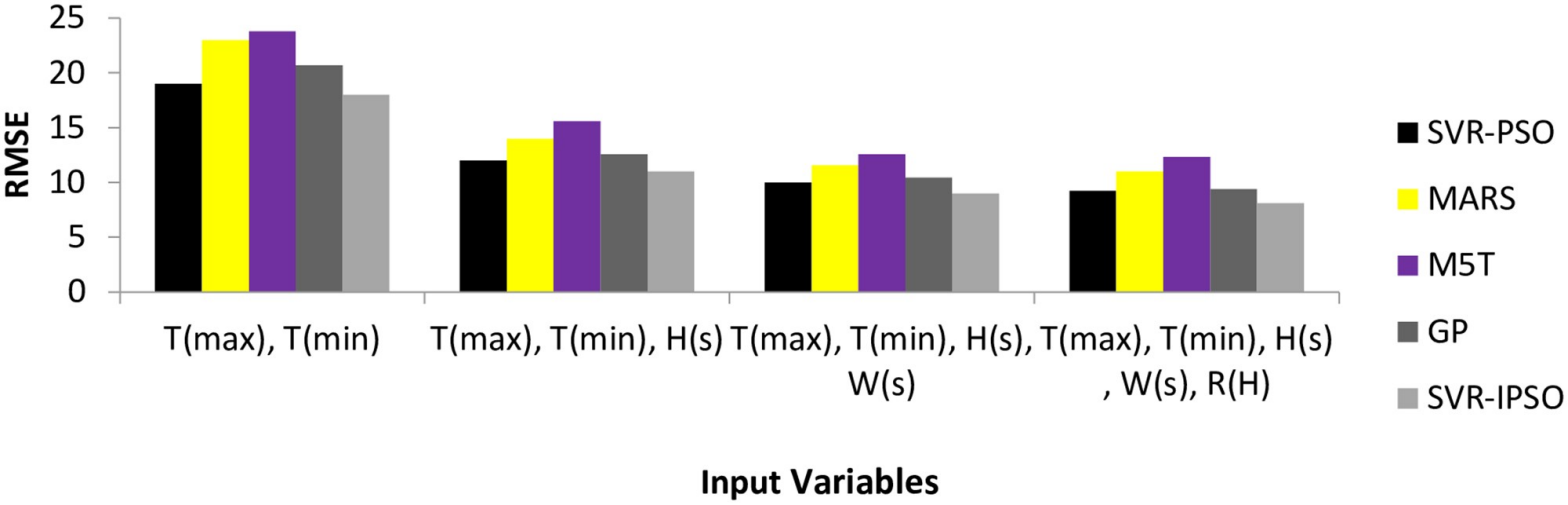
Computed RMSE for Antakya station.

Based on the comparison of the performances, as shown in the Table 2, it is noticeable that M5T (1) model has the lowest accuracy, with the first inputs. Thus, RMSE, MAE, NSE and MBE had the highest values in comparison to the SVR-PSO and MARS models. Increasing the number of inputs improved the performance of the M5T model for all indices. For example, the RMSE for M5T (4) was 48, 20 and 1.8% less than those for M5T (3), M5T (5) and M5T (1), respectively. Fig 4 shows the values of RMSE (the objective function) for different models.

**Fig 5:**
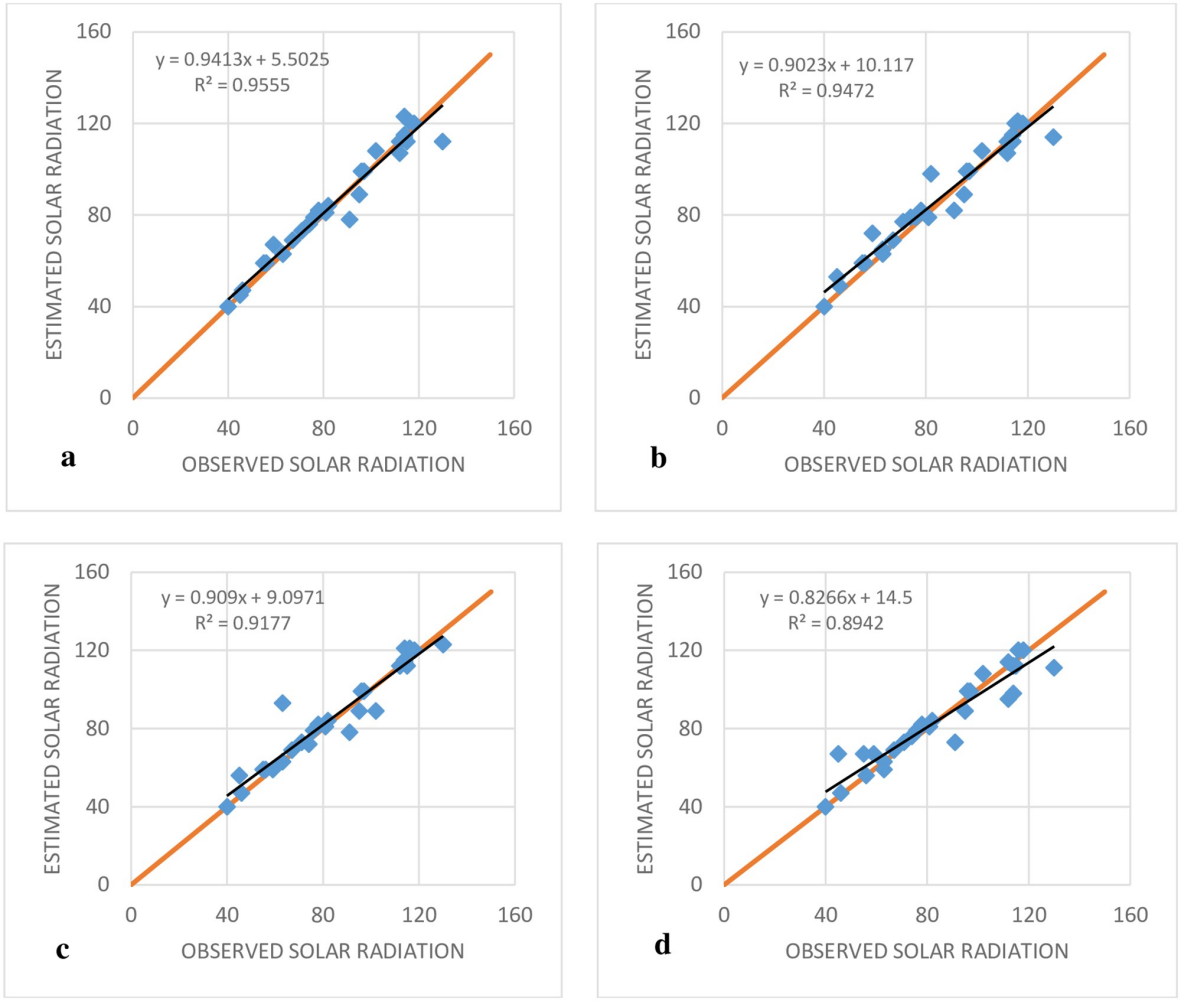
Observed and estimated SR of Antakya station at the test stage. (A) SVR-IPSO (4), (B) SVR- IPSO (3), (C) SVR-I PSO (2) and (D) SVR-IPSO (1).

The RMSE indices for the SVR-IPSO method, based on all inputs, were less than those for the MARS and M5T models (Fig 4). In addition, the lowest value of the RMSE index was exhibited by SVR-IPSO (4) and the highest value was exhibited by M5T (1). The general results for the Antakya station showed that SVR-PSO had the best performance in comparison to the other models. Fig 5 shows the R^2^ coefficients for different SVR-IPSO models. Based on its higher value for the R^2^ coefficient (Fig 5), SVR-IPSO (4) performed better than the other SVR-IPSO models. In general, the kind of inputs and the number of inputs affect the accuracy of the results. The consideration of parameters with high correlations, such as maximum and minimum temperature, can be good choices for inputs. In addition, one of the most important factors for the evaluation of the different methods is the computational time. Table 2 shows that SVR-IPSO could obtain the desired outputs with less computational time. For example, the computational time for SVR-IPSO (4) is, 5.2% 13, 24 and 5% less than those for SVR-PSO (4), M5T (4), MARS (4) and GP (4), respectively. The SVR-IPSO can improve the computational time because the convergence velocity is increased by this method.

### Adana station

Table 3 shows the performance of different models for the Adana station. All indices indicated that SVR-IPSO has a remarkable advantage over the MARS, SVR-PSO and M5T models. For example, the MAE for SVR-IPSO (4) was 47%, 19 and 11% less than those for SVR-IPSO (1), SVR-IPSO (2) and SVR-IPSO (3), respectively. Increasing the number of inputs for the Adana station improved the results, as for the Antakya station. For example, the RMSE for SVRI-PSO (4) was 10.02, approximately 52, 19 and 17% lower than those for SVR-IPSO (1), SVR-IPSO (2) and SVR-PISO (3), respectively. Other indices also indicated the superiority of SVR-IPSO (4) over other SVR-IPSO models.

**Table 3:** Comparison of statistical indices for different models for estimation of solar radiation in the test stage for Adana station.

Method	Input variables	MAE (Langley)	RMSE (Langley)	MBE	NSE	Time (s)
SVR-IPSO (1)	T_max_, T_min_	17.01	21.12	0.042	0.701	9
SVR-PSO (1) *κ* = 0.05, *C* = 69, *γ* = 0.117	T_max_, T_min_	17.54	22.12	0.045	0.699	10
M5T (1) [3]	T_max_, T_min_	21.39	26.54	0.053	0.611	12
MARS (1) [3]	T_max_, T_min_	19.67	23.46	0.049	0.696	14
GP (1)	T_max_, T_min_	17.61	22.02	0.042	0.612	11
SVR-IPSO (2)	T_max_, T_min_, H_s_	11.10	12.21	0.032	0.734	12
SVR-PSO (2) *κ* = 0.05, *C* = 62, *γ* = 0.167	T_max_, T_min_, H_s_	11.23	14.23	0.035	0.717	14
M5T (2) [3]	T_max_, T_min_, H_s_	15.65	19.29	0.061	0.795	16
MARS (2) [3]	T_max_, T_min_, H_s_	12.88	15.49	0.039	0.868	18
GP (2)	T_max_, T_min_, H_s_	11.36	14.35	0.037	0.719	15
SVR-IPSO (3)	T_max_, T_min_, H_s_, W_s_	10.12	12.01	0.032	0.761	15
SVR-PSO (3) *κ* = 0.05, *C* = 60, *γ* = 0.166	T_max_, T_min_, H_s_, W_s_	10.25	12.11	0.034	0.711	16
M5T (3) [3]	T_max_, T_min_, H_s_, W_s_	15.36	19.13	0.079	0.798	17
MARS (3) [3]	T_max_, T_min_, H_s_, W_s_	11.91	14.69	0.036	0.881	19
GP (3)	T_max_, T_min_, H_s_, W_s_	10.27	12.22	0.39	0.722	17
SVR-IPSO (4)	T_max_, T_min_, H_s_, W_s_, H_s_	9.01	10.02	0.034	0.871	17
SVR-PSO (4) *κ* = 0.09, *C* = 62, *γ* = 0.165	T_max_, T_min_, H_s_, W_s_, H_s_	9.87	10.32	0.044	0.709	18
M5T (4) [3]	T_max_, T_min_, H_s_, W_s_, R_h_	15.97	20.89	0.072	0.759	20
MARS (4) [3]	T_max_, T_min_, H_s_, W_s_, R_h_	12.79	15.40	0.021	0.869	22
GP (4)	T_max_, T_min_, H_s_, W_s_	9.99	10.35	0.046	0.712	24

The SVR-IPSO, SVR-PSO, MARS and M5T models were compared for the Adana station. Multiple indices indicated the superiority of the SVR-IPSO model over the SVR-PSO MARS and M5T models. For example, the M5T (3) model had the lowest MAE (15.36) in comparison to the other M5T models, and the MARS (3) model exhibited the best performance of the MAE index (11.91) in comparison to the other MARS models. However, the MAE values for these two models were 36 and 18% more than SVR-PSO (4). Other indices also indicated the superiority of SVR-IPSO (4) over the MARS and M5T models. However, a comparison of the results for GP and SVR-IPSO shows better performance for GP. For example, the MAE and RMSE values for SVR-IPSO (4) are 1.20 and 3.1% less than those for GP (4). In addition, the GP model has the best performance in comparison to the MARS and M5T models. Increasing the inputs for the different models shows that models with 5 inputs have the best performance.

**Fig 6:**
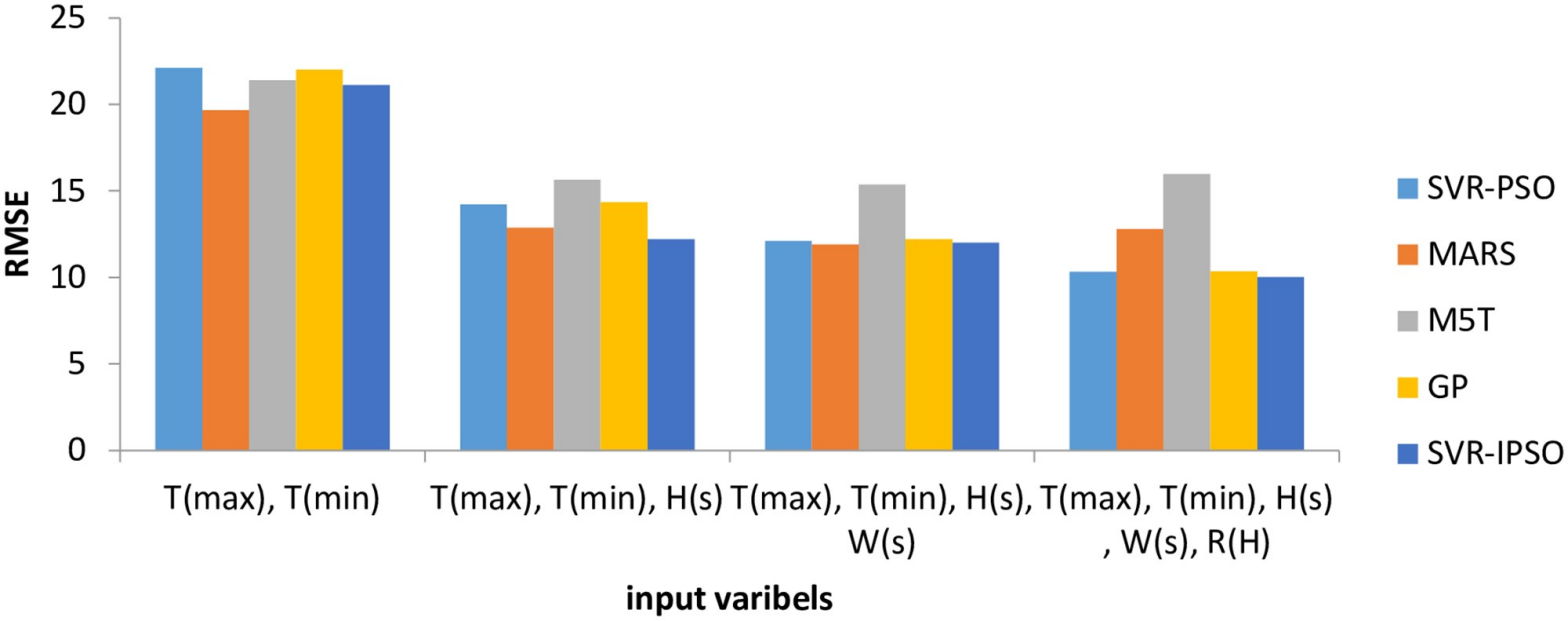
Computed RMSE for Adana station.

**Fig 7:**
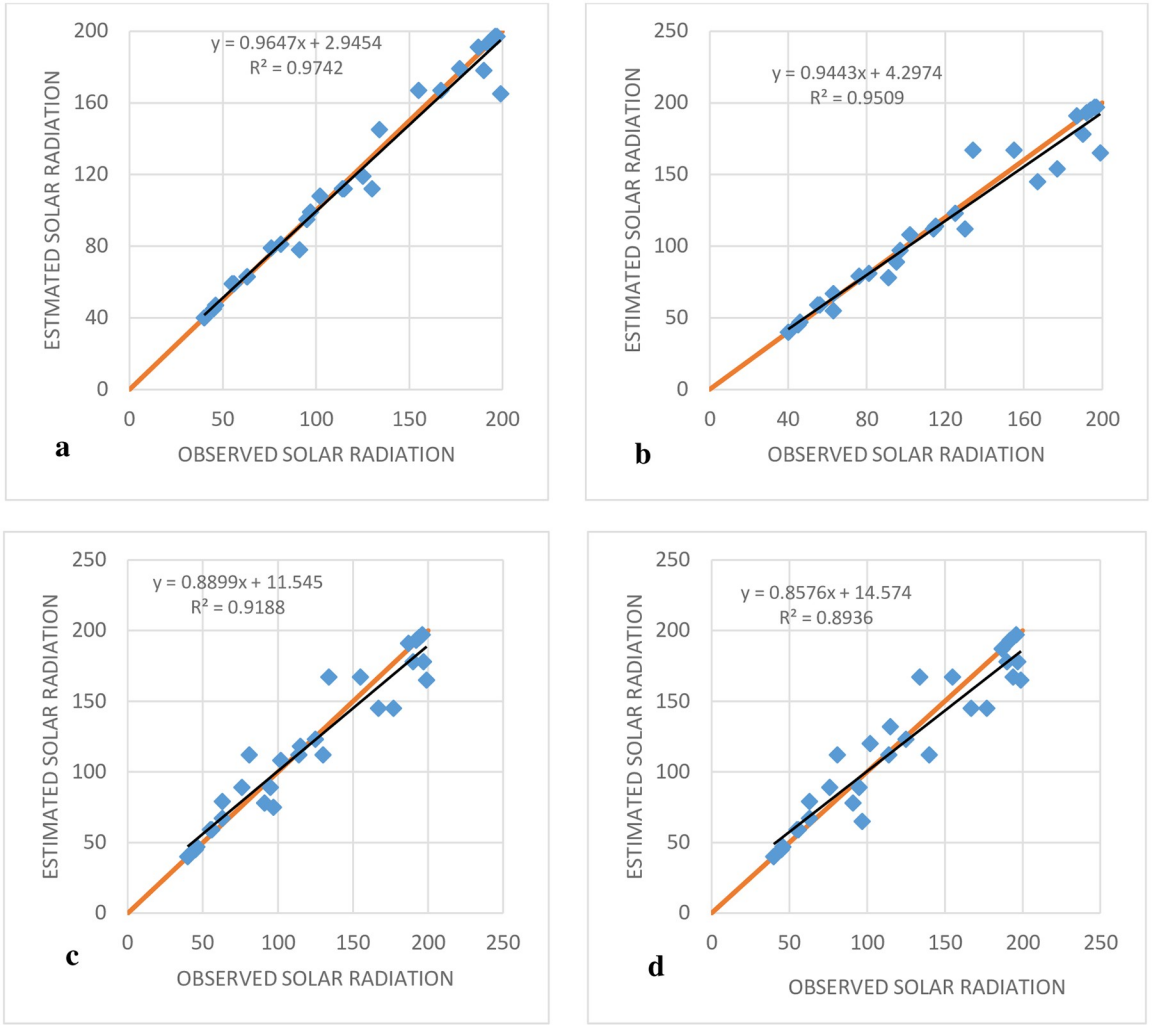
Observed and estimated SR of Adana station at the test stage. (A) SVR-IPSO (4), (B) SVR- IPSO (3), (C) SVR- IPSO (2) and (D) SVR- IPSO (1).

The various indices did not unanimously indicate the superiority of a specific model as the best among the M5T models. For example, based on RMSE and MAE, M5T (3) had the best performance in comparison to the other M5T models. However, the MBE showed better performance for M5T (1) in comparison to the other M5T models. Fig 6 shows the performance of different models based on the RMSE (objective function) for the Adana station. The SVR-IPSO models for all input combinations had the best performance in comparison to the SVR-PSO, MARS and M5T models. The lowest value for RMSE was exhibited by SVR-IPSO (4) and the worst performance was exhibited by M5T (4) (Fig 6). Fig 7 shows the R^2^ coefficients for the SVR-IPSO models. The results showed that SVR-IPSO (4) performed better than the other SVR-IPSO models based on its higher value for the R^2^ coefficient (Fig 7). In addition, the computational times in Table 3 show that SVR-IPSO performed better than the other methods.

### Periodic model for computing solar radiation at the Antakya station

A periodic forecast entails the addition of the months as an input. Many studies have shown that periodic prediction can improve the results of precise forecasts of hydrological and climatic data. All simulation models in Table 4 are based on the addition of the month as an input. The results in Table 4 tangibly show the superiority of the SVR-IPSO model over the other models. For example, the MAE index indicated that SVR-IPSO (4), SVR-IPSO (3), M5T (4) and MARS (4) were the best in comparison to the other SVR-IPSO, SVR-PSO, M5T and MARS models. The MAE for SVR-IPSO (4) was 4.01; this was 26, 6.7 and 1.3% better than those of the M5T (4), SVR-PSO and MARS (4) models and showed the superiority of SVR-IPSO (4) over the other models. The NSE index also indicated the superiority of SVR-IPSO over the other models. For example, the highest value for the NSE (0.911) was exhibited by SVR-PSO (4), while the best values of this index for the M5T and MARS models were 0.963 and 0.981, respectively. In addition, a comparison of these results with the GP model shows that SVR-IPSO has the best performance; the error indices of SVR-IPSO are less than those of GP. For example, the MAE values for SVR-IPSO (4), SVR-IPSO (3), SVR-IPSO (2) and SVR-IPSO (1) are 7.1, 0.92, 2.8% and 1.3% less than those for GP (1), GP (2), GP (3), and GP (4), respectively. Increasing the number of inputs has a good effect on the simulation results; the fourth combination of each methods exhibits the best results. This shows that all the parameters, namely the maximum temperature, minimum temperature, wind speed, relative humidity and month, affect the results.

**Table 4:** Comparison of statistical indices for different methods for estimation of solar radiation at the test stage based on periodic models for Antakya station.

Method	Input variables	MAE	RMSE	MBE	NSE	Time (s)
SVR-IPSO (1)	T_max_, T_min_, month	6.40	7.20	0.065	0.961	19
SVR-PSO (1) *κ* = 0.05, *C* = 60, *γ* = 0.165	T_max_, T_min_, month	6.45	7.23	0067	0.959	20
M5T (1) [3]	T_max_, T_min_, month	10.06	12.1	0.091	0.954	22
MARS (1) [3]	T_max_, T_min_, month	6.83	8.48	0.077	0.906	24
GP (1)	T_max_, T_min_, month	6.49	7.29	0.069	0.961	21
SVR-IPSO (2)	T_max_, T_min_, H_s_, month	6.05	7.10	0.060	0.955	20
SVR-PSO (2) *κ* = 0.04, *C* = 60, *γ* = 0.166	T_max_, T_min_, H_s_, month	6.20	7.12	0.063	0.962	22
M5T (2) [3]	T_max_, T_min_, H_s_, month	11.8	11.8	0.090	0.912	24
MARS (2) [3]	T_max_, T_min_, H_s_, month	8.18	8.18	0.085	0.957	26
GP(2)	max, Tmin, Hs, month	6.23	7.91	0.089	0.967	23
SVR-IPSO (3)	T_max_, T_min_, H_s_, W_s_, month	5.28	5.40	0.001	0.986	24
SVR-PSO (3) *κ* = 0.05, *C* = 54, *γ* = 0.166	T_max_, T_min_, H_s_, W_s_, month	5.34	5.45	0.002	0.981	25
M5T (3) [3]	T_max_, T_min_, H_s_, W_s_, month	7.63	7.63	0.003	0.963	27
MARS (3) [3]	T_max_, T_min_, H_s_, W_s_, month	5.83	5.83	0.029	0.978	29
GP (3)	Tmax, Tmin, Hs, Ws, month	5.39	5.55	0.002	0.979	26
SVR-IPSO (4)	T_max_, T_min_, H_s_, W_s_, R_h_, month	4.01	5.02	0.002	0.991	26
SVR-PSO (4) *κ* = 0.05, *C* = 62, *γ* = 0.167	T_max_, T_min_, H_s_, W_s_, R_h_, month	4.30	5.12	0.002	0.985	28
M5T (4) [3]	T_max_, T_min_, H_s_, W_s_, R_h_, month	5.87	7.68	0.005	0.962	30
MARS (4) [3]	T_max_, T_min_, H_s_, W_s_, R_h_, month	4.36	5.42	-0.019	0.981	32
GP (4)	Tmax, Tmin, Hs, Ws, Rh, month	4.32	5.18	0.004	0.989	29

**Fig 8:**
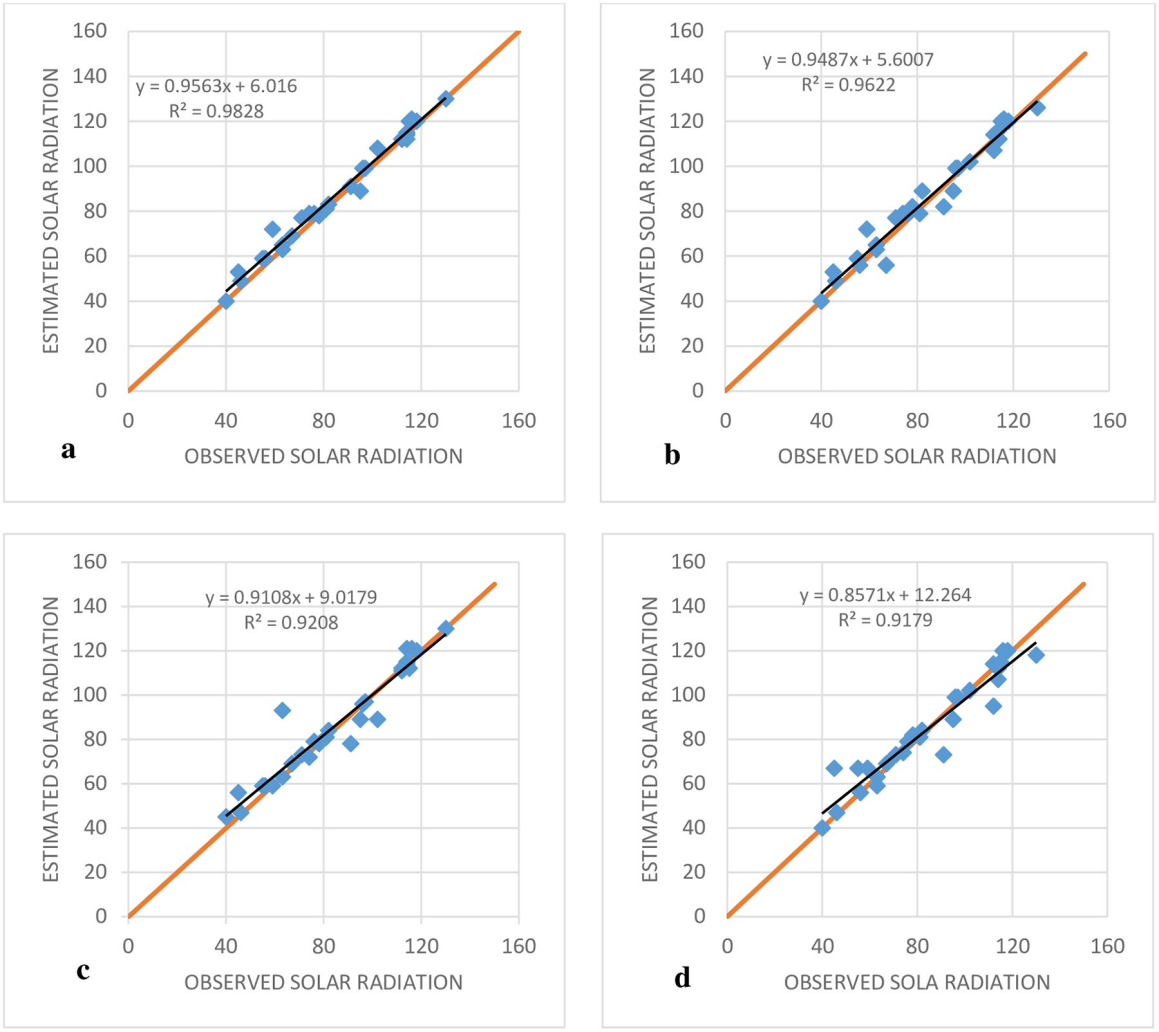
Observed and estimated SR of Antakya station at test stage based on the periodic model. (A) SVR-IPSO (4), (B) SVR- IPSO (3), (C) SVR- IPSO (2) and (D) SVR- IPSO (1).

The trend of the results showed that adding inputs improved the performance of SVR-IPSO for all indices; this was not true for the M5T and MARS models. Another important point was a comparison of the forecasts with/without adding the month. For example, the RMSE for SVR-IPOS (4) based on the periodic model and Table 4 was 5.02, while it was 9.01 based on Table 2 without adding the month as the input. Other indices for the other models indicated that the value of the error indices could be significantly reduced by adding the month as an input. For example, the NSE index showed that, assuming periodic simulation, the M5T (3) model had the best performance in comparison to the other M5T models; the value of the index for this model was 0.963, while the value of the index for the best M5T model without adding the month was 0.903 based on Table 2. Thus, prediction can improve the results for all models. Fig 8 shows the R^2^ coefficients for the SVR-IPSO models. Fig 8 indicates that the periodic SVR-IPSO models perform better than the non-periodic models shown in Fig 5. In addition, the trend in computational time for the different methods shows that increasing the number of inputs increases the computational time and that SVR-IPSO has the best performance in comparison to the other methods.

### Periodic model for computing solar radiation at the Adana station

Table 5 shows the performance of different models with periodic prediction for the Adana station. The MAE for SVR-IPSO (4) was 4.12, which was 30, 37 and 57% less than those for SVR-IPSO (3), SVR- IPSO (2) and SVR-IPSO (1), respectively. Based on MAE and Table 6, M5T (3), SVR-PSO (4) and MARS (2) performed better than the other MARS and M5T models. The MAE for SVR-IPSO (4) was 37, 47 and 42% less than those for the, SVR-PSO (4), M5T (3) and MARS (2) models, indicating the superiority of the SVR-IPSO model. A reduction in the error indices can be achieved by increasing the number of inputs for the SVR-PSO model, as in the previous sections. The NSE for SVR-IPSO (4) was 0.992, while the best values of this coefficient for MARS and M5T were 0.942 and 0.942; this indicates the superiority of the SVR-IPSO model over the other models. In addition, the results show that the GP model performs better than MARS and M5T, but SVR-IPSO performs better than GP. For example, the RMSE for SVR-IPSO (4), SVR-IPSO (3), SVR-IPSO (2) and SVR-IPSO (1) were 12, 14, 3.2 and 2.6% less than those for GP (4), GP (3), GP (2) and GP (1), respectively.

**Table 5:** Comparison of statistical indices for different models for estimation of solar radiation at the test stage based on periodic models for Adana station.

Method	Input variables	MAE	RMSE	MBE	NSE	Time (s)
SVR-IPSO (1)	T_max_, T_min_, month	9.52	10.82	-0.012	0.878	24
SVR-PSO (1) *κ* = 0.05, *C* = 60, *γ* = 0.166	T_max_, T_min_, month	9.54	10.9	-0.014	0.933	25
M5T (1) [3]	T_max_, T_min_, month	10.2	13.2	-0.016	0.903	27
MARS (1) [3]	T_max_, T_min_, month	9.17	11.2	-0.031	0.930	29
GP (1)	Tmax, Tmin, month	9.59	11.12	-0.015	0.932	26
SVR-IPSO (2)	T_max_, T_min_, H_s_, month	6.56	8.84	-0.090	0.962	25
SVR-PSO (2) *κ* = 0.05, *C* = 62, *γ* = 0.165	T_max_, T_min_, H_s_, month	7.01	9.12	-0.010	0.950	27
M5T (2) [3]	T_max_, T_min_, H_s_, month	9.26	9.66	-0.011	0.924	28
MARS (2) [3]	T_max_, T_min_, H_s_, month	7.10	11.72	-0.026	0.949	30
GP (2)	T_max_, T_min_, H_s_, month	7.05	9.14	0.011-	0949	29
SVR-IPSO (3)	T_max_, T_min_, H_s_, W_s_, month	5.89	8.02	-0.009	0.962	27
SVR-PSO (3) *κ* = 0.05, *C* = 60, *γ* = 0.166	T_max_, T_min_, H_s_, W_s_, month	6.94	9.01	-0.010	0.951	28
M5T (3) [3]	T_max_, T_min_, H_s_, W_s_, month	7.79	10.3	-0.015	0.942	31
MARS (3) [3]	T_max_, T_min_, H_s_, W_s_, month	7.68	10.3	-0.037	0.942	33
GP (3)	T_max_, T_min_, H_s_, W_s_, month	6.99	9.23	-0.010	0.949	29
SVR-IPSO (4)	T_max_, T_min_, H_s_, W_s_, R_h_, month	4.12	7.64	-0.008	0.992	29
SVR-PSO (4) *κ* = 0.05, *C* = 60, *γ* = 0.166	T_max_, T_min_, H_s_, W_s_, R_h_, month	6.45	8.67	-0.009	0.970	30
M5T (4) [3]	T_max_, T_min_, H_s_, W_s_, R_h_, month	8.46	10.40	-0.032	0.940	32
MARS (4) [3]	T_max_, T_min_, H_s_, W_s_, R_h_, month	7.84	10.1	-0.018	0.944	33
GP (4)	T_max_, T_min_, H_s_, W_s_, R_h_, month	6.49	8.69	-0.009	0.971	31

**Table 6:** Comparison of statistical indices for different methods for estimation of daily solar radiation in Konay station.

Method	Input variables	MAE%	RMSE%	MBE%	NSE	Time (s)
SVR-IPSO (1)	T_max_, T_min_	12	14	8	0.84	15
SVR-PSO (1)	T_max_, T_min_	14	16	9	0.80	18
SVR-FFA (1)	T_max_, T_min_	17	20	10	0.79	22
SVR-GA (1)	T_max_, T_min_	22	23	12	0.77	25
SVR-IPSO (2)	T_max_, T_min_, H_s_	10	12	7	0.89	17
SVR-PSO (2)	T_max_, T_min_, H_s_	12	14	8	0.83	20
SVR-FFA. (2)	T_max_, T_min_, H_s_	15	18	10	0.82	25
SVR-GA (2)	T_max_, T_min_ `, H_s_	16	21	11	0.81	27
SVR-IPSO (3)	T_max_, T_min_, H_s_, W_s_	9	10	5	0.91	19
SVR-PSO (3)	T_max_, T_min_, H_s_, W_s_	11	12	7	0.85	21
SVR-FFA (3)	T_max_, T_min_, H_s_, W_s_	12	16	8	0.83	26
SVR-GA (3)	T_max_, T_min_, H_s_, W_s_	14	18	9	0.82	28
SVR-IPSO (4)	T_max_, T_min_, H_s_, W_s_, Rh	7	9	3	0.95	20
SVR-PSO (4)	T_max_, T_min_, H_s_, W_s_, Rh	10	10	5	0.90	22
SVR-FFA (4)	T_max_, T_min_, H_s_, W_s_, R_h_	11	14	6	0.88	27
SVR-GA (4)	T_max_, T_min_, H_s_, W_s_, R_h_	12	16	7	0.86	30

In addition, periodicity improved the results. For example, the RMSE for SVR-PSO (4) without periodicity, according to Table 3, was 10.02, while it was 7.64 for the periodic SVR-IPSO (4) model. Other indices for other models indicated similar effects. For example, M5T (2) exhibited the lowest value for RMSE in comparison to the other M5T models (based on Table 5), which was 9.66. On the other hand, the value of RMSE for the best model without periodicity (M5T), based on Table 4, was 19.13. Thus, it is obvious that periodicity improved the results. Furthermore, the results show that the addition of the month as an input resulted in the best outcome for GP; the index error was reduced in comparison to the simulation results for which the month was not an input. In addition, SVR-IPSO exhibited decreased computational time.

**Fig 9:**
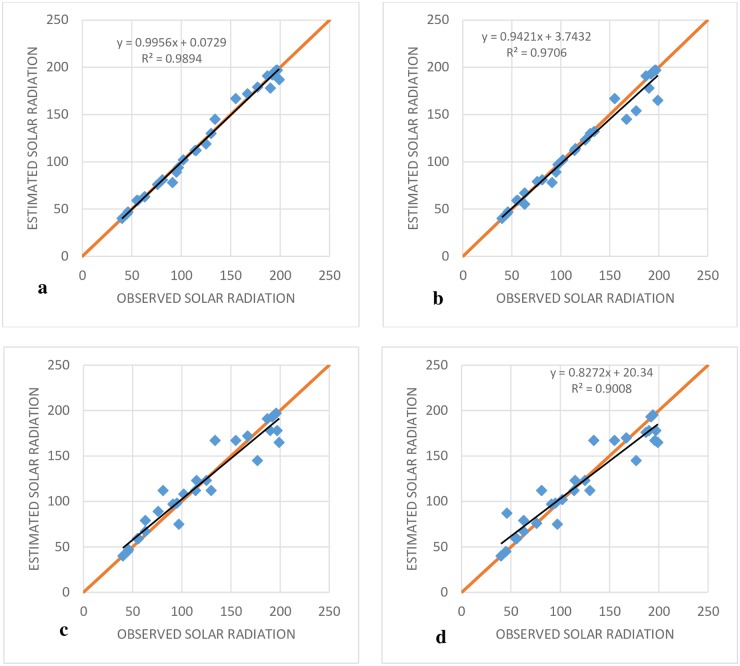
Observed and estimated SR of Adana station based on periodic predication at the test stage. (A) SVR-IPSO (4), (B) SVR- IPSO (3), (C) SVR- IPSO (2) and (D) SVR- IPSO (1).

Fig 9 shows the R^2^ coefficients for different SVR-IPSO models. SVR-IPSO (4) has the highest value; furthermore, the values of R^2^ for the models in Fig 7 was better than those for the models in Fig 9 (without periodic prediction).

The results indicated that the SVR-IPSO act better than other models. There is more challenge for solar radiation estimation such as daily solar radiation or solar radiation for different climates. Thus, the combination of Tables 2 and 3 were used to estimate daily solar radiation for better evaluation of new model. Also, previous studies suggested that the SVR with the other optimization algorithms. Genetic algorithm and firefly algorithms were used to determine the SVR parameters. The firefly algorithm (FFA) acts based on social behavior in the fireflies. The attractiveness of each firefly is based on its light intensity. The light intensity for each firefly is considered as objective function and the social behavior for the fireflies is used for the optimization algorithm. More details can be seen in the [33]. Also, the genetic algorithm acts based on natural selection and chromosomes. The mutation and crossover and selection operators were used to improve the solutions and more details can be seen in the [47]. Thus, the SVR-IPSO, SVR-PSO, SVR-FFA and SVR–GA are used for the estimation of daily solar radiation for different station with different climatic condition. Konya station is selected as station for evaluating of validation of SVR-IPSO. This station Konya is located at the 37°58′*N*, 32°32′*E* in a semi dry climate. Also, the average temperature for this station is 23.3°C. There are 2860 sunshine duration per year averagely. The estimation of daily solar estimation for period 1981 to 2016 is considered as more challenging for the SVR-IPSO model.

The RMSE, MAE and MBE index are computed on percentage. Table 6 shows the daily solar radiation for Konya Station 1981 to 2016. The results indicated that the SVR-IPSO has the less RMSE and MAE value and thus, the fourth combination for SVR-IPSO is better compared to the SVR-GA, SVR-PSO and SVR-FFA. Also, the worst performance is also observed and recorded for the SVR-GA. The first input for the SVR-GA has the worst performance compared to SVR-GA (2), SVR-GA (3) and SVR-GA (4). The NSE value for SVR-IPSO (4) has the most value compared to the other model and least value for the NSE is related to the SVR-GA. However, the results indicate that the SVR-IPSO can shows a good performance for different climates for daily or monthly solar estimation.

It is true that the accuracy of the any forecasting model will be affected positively or negatively, when using future input data (in this study metrological data). Therefore, there is a need to keep updating the forecasting model by feeding it with the new patterns of the future data. However, it should be noted that as long as the future data is similar with the used data pattern, the model’s performance will not change much. Based on the RMSE for the best model (SVR-IPSO (4)), the enhancement rate has been investigated by comparing with different studies in the literature. Table 7 summarize the enhancement that has been accomplished by the proposed model for two stations (Adana and Antakya). The proposed model shows a significant enhancement in the performance for both stations. It can be determined that the significant of performance is (42.12%–20.91%) for Adana and (58.51%–7.38%) for Antakya.

**Table 7:** The enhancement rate for best model (SVR-IPSO (4)) comparing with the previous study [3].

Method	Enhancement Rate	
	Adana	Antakya
M5T (1) [3]	42.12%	58.51%
MARS (1) [3]	31.78%	40.80%
M5T (2) [3]	20.91%	57.45%
MARS (2) [3]	34.81%	38.63%
M5T (3) [3]	25.82%	34.20%
MARS (3) [3]	25.82%	13.89%
M5T (4) [3]	26.53%	34.63%
MARS (4) [3]	24.35%	7.38%

## Conclusion

The present study introduced a new prediction model for SR. The model is essentially based on an improved SVR integrated withI PSO;I PSO determines the optimum values of the unknown SVR parameters. The proposed model was applied to two stations from Turkey for evaluation against the previously developed SVR-PSO, MARS, GP and M5T models, which have been applied to the same stations. Based on the proposed performance indicators, increasing the number of inputs improved the results of the SVR-IPSO model. In addition, the application of SVR-IPSO to the Antakya station showed the superiority of SVR-IPSO over the other models. The proposed SVR-IPSO models for the two stations achieved better performance than the MARS, GP, SVR-PSO and M5T models for different input scenarios. Furthermore, an additional input variable representing the month of the year resulted in improvements over previous input scenarios. In conclusion, the proposed SVR integrated with IPSO (SVR-IPSO) can be considered an effective tool for solar radiation prediction that could help decision-makers create efficient plans for renewable energy production. A few important variables were lacking in the selected stations and hence could not be examined in this study. Also, the SVR-IPSO, was validated for Konya station and the results were compared with the SVR-GA, SVR-PSO and SVR-FFA. The results showed that the SVR-IPSO model has best performance comparing with all the presented models.

The proposed model could be improved by adding other input variables that directly influence solar radiation. Future studies should consider additional input variables that might improve the accuracy of predicting solar radiation. In addition, an integration of the SVR and advanced meta-heuristic optimization algorithms should be investigated as it might improve the forecasting accuracy for SR. The sun radiation has a direct effect on the climate condition therefore, it is essential to consider further evaluation for the proposed model in different climatic zone.
